# Supplementary material for: ExoSTING, an extracellular vesicle loaded with STING agonists, promotes tumor immune surveillance
Source: Commun Biol. 2021 Apr 22;4:497. doi: 10.1038/s42003-021-02004-5 (PMC8062530; doi:10.1038/s42003-021-02004-5)
Supplement: Supplementary file 2 — Supplementary Information [file 42003_2021_2004_MOESM2_ESM.pdf]

Supplementary Information

ExoSTING, an extracellular vesicle loaded with STING agonists, promotes tumor immune surveillance

Su Chul Jang[1], Kyriakos D. Economides[1], Raymond J. Moniz[1], Chang Ling Sia[1], Nuruddeen Lewis[1], Christine McCoy[1], Tong Zi[1], Kelvin Zhang[1], Rane A. Harrison[1], Joanne Lim[1], Joyoti Dey[2], Marc Grenley[2], Katherine Kirwin[1], Nikki Ross[1], Raymond Bourdeau[1], Agata Villiger-Oberbek[1], Scott Estes[1], Ke Xu[1], Jorge Sanchez-Salazar[1], Kevin Dooley[1], William K. Dahlberg[1], Douglas E. Williams[1], Sriram Sathyanarayanan[1*]

[1] Codiak BioSciences Inc., Cambridge, Massachusetts, USA

[2] Presage Biosciences, Seattle, Washington, USA.

*To whom correspondence should be addressed: S.S. (sriram.sathy@codiakbio.com).

Supplementary Table 1: Pharmacokinetic Parameters in Injected Tumor Samples

**Parameter**	**exoCDN2 (0.3 µg)**	**CDN2 (0.3 µg)**	**CDN2 (30 µg)**
C_{max} (ng/mL)	1,790	1,240	13,800
AUC (ng·hr/mL)	7,510	799	97,300
$T_{1/2}$ (hours)	2.9	0.620	2.05
CL (mL/hr)	0.0399	0.375	0.308

AUC=area under the concentration-time curve; CL=clearance; C_{max}=maximum concentration; $T_{1/2}$=half-life

Supplementary Table 2: Antibodies used in studies.

Antibody	Vendor	Part #	Additional information
**Western blotting**			**Blotting conditions**
Human anti-CD9	BioLegend	312102	1:1000 Dilution, Non-reduced
Human anti-CD63	ThermoFisher	10628D	1:1000 Dilution, Non-reduced
Human anti-CD81	ThermoFisher	10630D	1:1000 Dilution, Non-reduced
Human anti-TSG101	BD Biosciences	612696	1:1000 Dilution, Reduced
Human anti-Alix	Abcam	ab117600	1:1000 Dilution, Reduced
Human anti-Calnexin	Abcam	ab22595	1:1000 Dilution, Reduced
Anti-Adenovirus Type 5 E1A antibody	Abcam	ab204123	1:2000 Dilution, Reduced
Human anti-PTGFRN	LS-Bio	LS-C158765	1:1000 Dilution, Reduced
Rabbit anti-Mouse IgG HRP	Abcam	ab97046	1:10000 Dilution
Goat anti-Rabbit IgG HRP	Abcam	ab6721	1:5000 Dilution
**Flow cytometry**			**Conjugates**
Mouse anti-CD45	BD Biosciences	564279	BUV395
Mouse anti-CD3e	BD Biosciences	564618	BUV737
Mouse anti-TCRb	BD Biosciences	564799	BUV737
Mouse anti-CD11b	BioLegend	101212	APC
Mouse anti-CD8	BD Biosciences	566409	BB700
Mouse anti-Ly-6C	BioLegend	128018	PE-Cy7
Mouse anti-I-A/I-E	BioLegend	107635	BV510
Mouse anti-CD335	BioLegend	137619	BV605
Mouse anti-CD49b	BD Biosciences	740363	BV605
Mouse anti-CD11c	BioLegend	117330	BV421
Mouse anti-XCR1	BioLegend	148206	APC
Mouse anti-F4/80	BioLegend	123141	BV785
Human anti-CD69	BioLegend	310912	PE-Cy7
Human anti-CD86	BioLegend	305412	APC
Human anti-CD11c	BioLegend	301628	BV421
Human anti-CD1C	BioLegend	331536	BV711
Human anti-CD14	BioLegend	367126	BV605
Human anti-CD123	BioLegend	306032	BV785
Human anti-CD141	BioLegend	344110	PE-Cy7

**Antibody**	**Vendor**	**Part #**	**Additional information**
Human anti-CD3	BD Biosciences	564117	BUV395
Human anti-CD19	BioLegend	302212	APC
Human anti-CD20	BD Biosciences	564432	BUV737
Human anti-CD16	BioLegend	302016	PE-Cy7
Human anti-CD56	BioLegend	318326	PerCP-Cy5.5
Human anti-HLADR	BioLegend	307606	PE
**Immunohistochemistry**			
Mouse anti-CD8	Abcam	ab230156	
Mouse anti-F4/80	Cell Signaling	70076s	
Mouse anti-phospho TBK1	Cell Signaling	5483	
Mouse anti-cleaved caspase 3	Cell Signaling	9661	

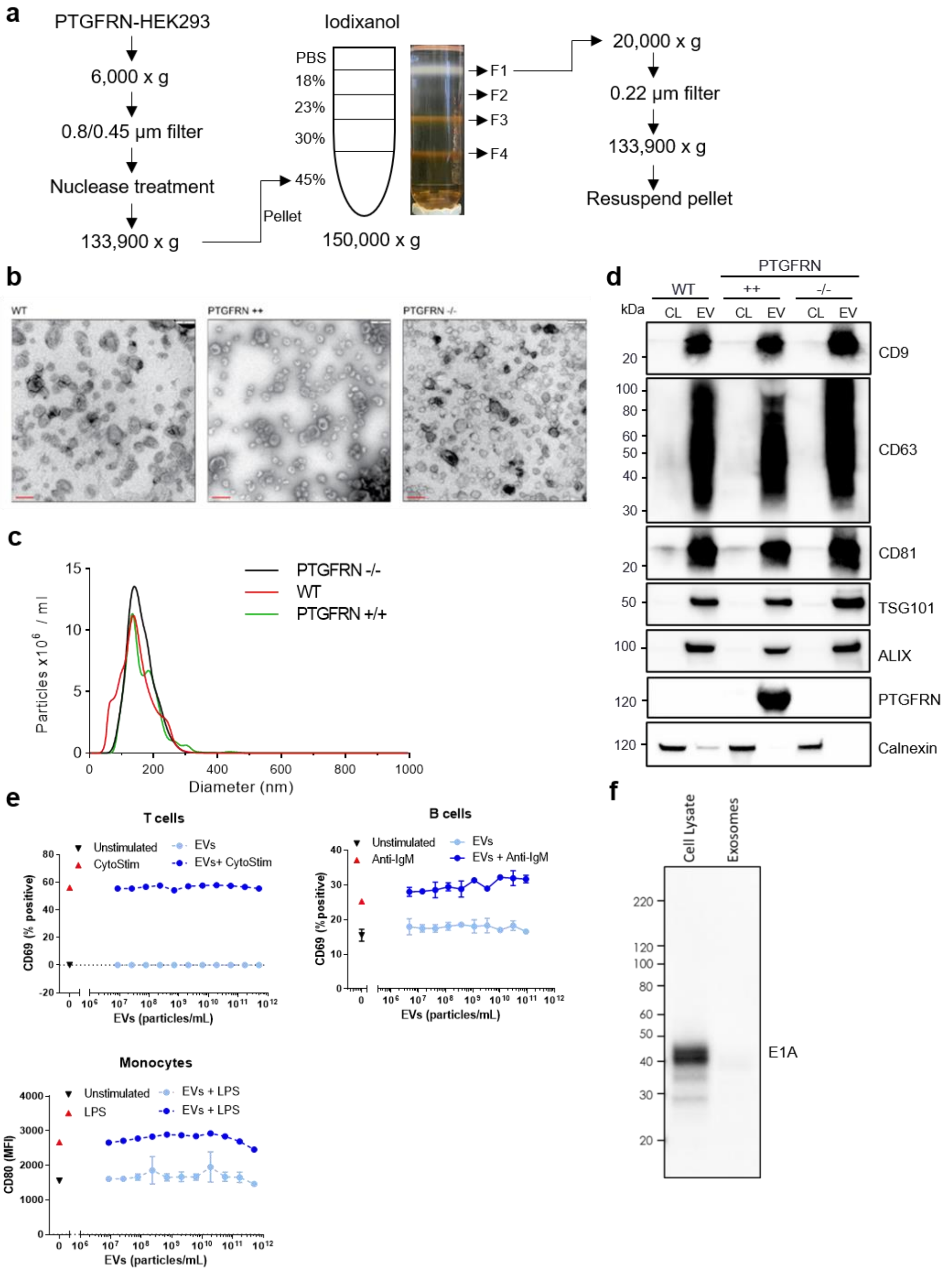

Supplementary Fig. 1: Characterization of EVs. a Schematic diagram of EV purification. **b** TEM images of PTGFRN-/-, wild-type (WT), and PTGFRN+/+ EVs. Scale bars, 200 nm. **c** Representative size distribution of PTGFRN-/-, WT, and PTGFRN+/+ EVs, measured by NTA. **d** Immunoblots of PTGFRN, Calnexin, and EV markers, CD9, CD63, CD81, Alix, and TSG101. SM; size marker, CL: cell lysate, EV; extracellular vesicles. **e** Representative dose-response curves (n = 2 healthy donors) of CD69 MFI on T cells and B cells and CD80 MFI on monocytes after treating with EVs (n = 2 biological replicates per donor). Data are presented as means ± s.d. from replicate samples. **f** Immunoblots of E1A protein on HEK293 cell lysate and EVs.

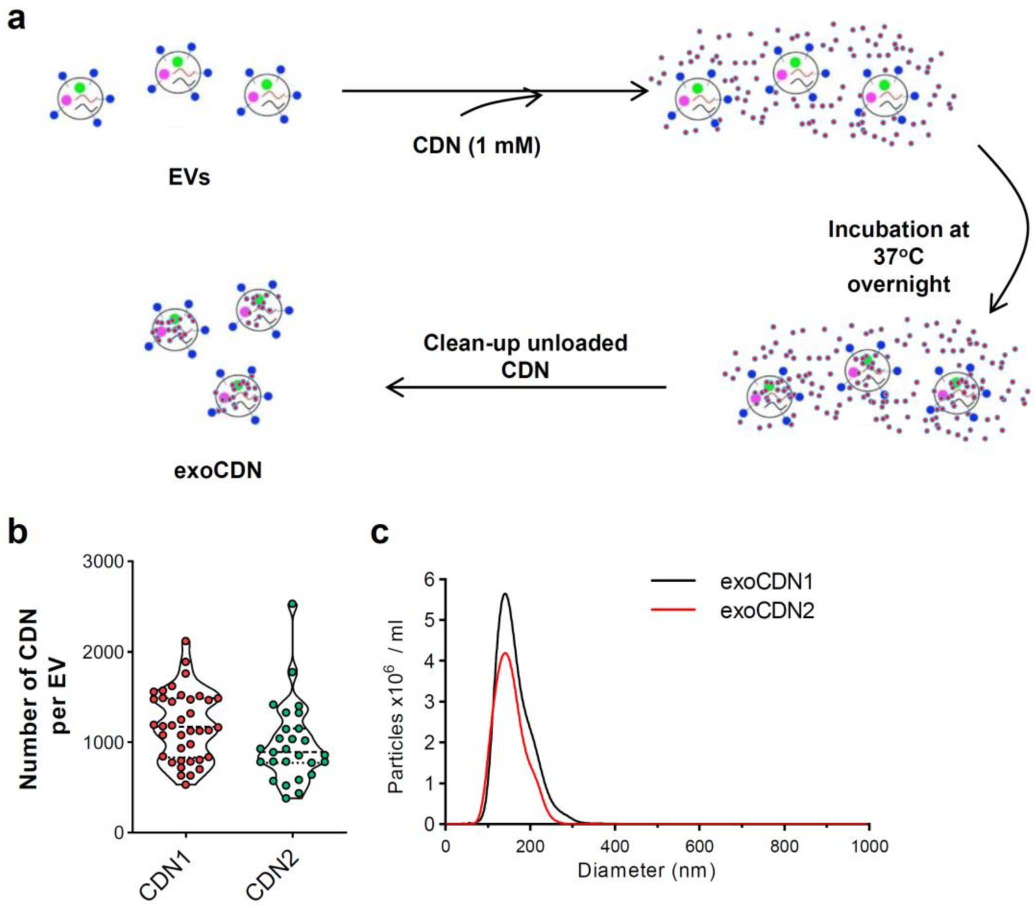

Supplementary Fig. 2: Loading and characterization of exoSTING. a Schematic illustration of loading process. **b** Number of CDN per EV after loading of two different CDNs (CDN1 and CDN2). CDNs were quantified by LC-MS/MS (*n* = 38 independent replicates for CDN1 and *n* = 27 independent replicates for CDN2). **c** Representative size distribution of exoCDN1 and exoCDN2, measured by NTA.

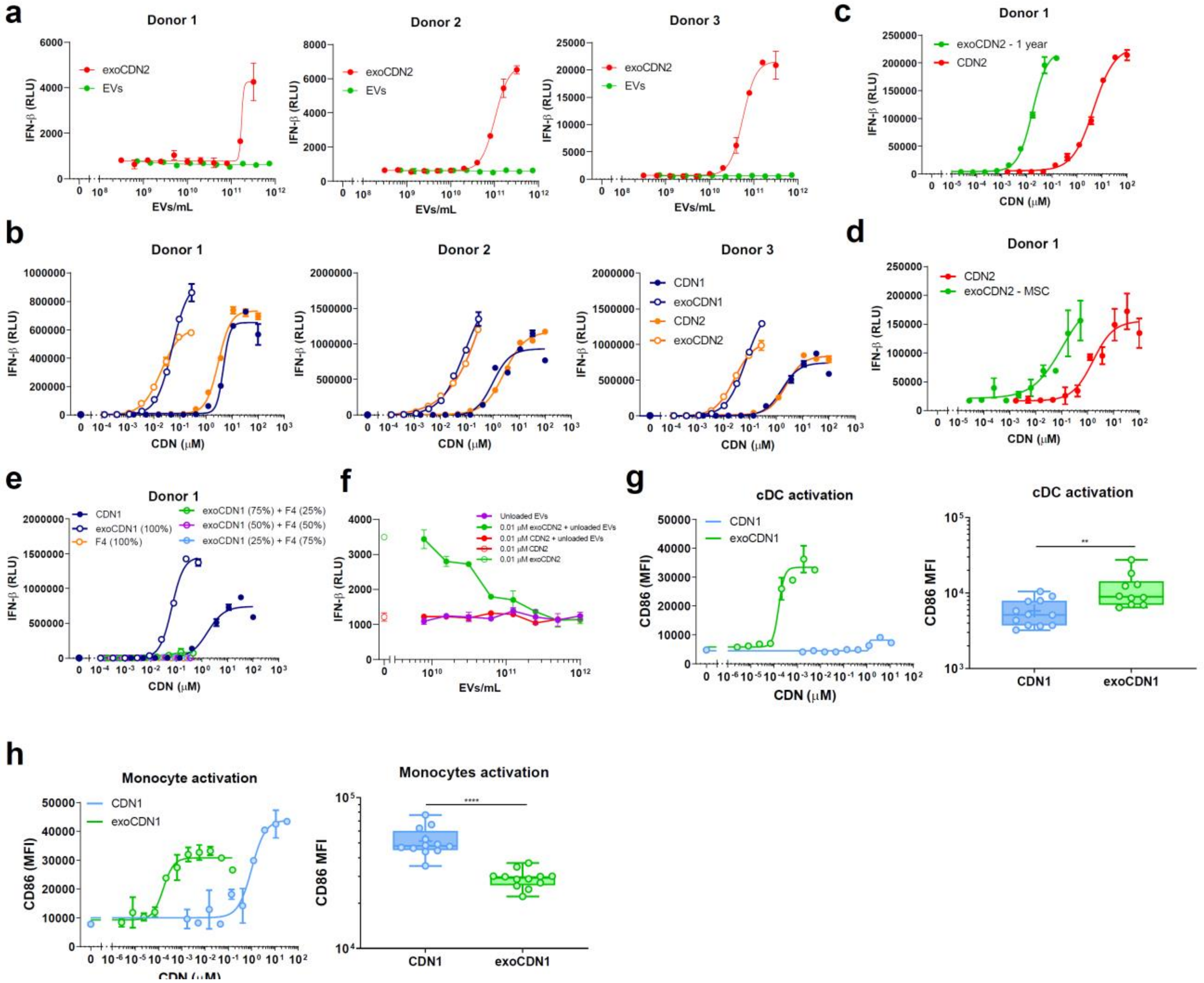

Supplementary Fig. 3: exoSTING enhanced *in vitro* potency of CDNs. a-c Dose response curves of IFN-β production in human PMBC supernatant after treating with two different CDN-loaded EVs, exoCDN1 and exoCDN2, or unloaded EVs, comparing with free CDNs (n = 2 biological replicates per donor). **d** Representative dose-response curves (n = 2 healthy donors) of IFN-β production in human PMBC supernatant after treating with exoCDN2 that derived from mesenchymal stem cells (MSC), comparing with free CDN (n = 2 biological replicates per donor). **e** Representative dose-response curves (n = 2 healthy donors) of IFN-β production in human PMBC after treating with exoCDN1 and different ratio of F4 impurity from Iodixanol gradient, loaded with CDN1 (Supplementary Fig. 1a) (n = 2 biological replicates per donor). **f** Representative dose-response curves (n = 2 healthy donors) of IFN-β production in human PMBC supernatant after co-treatment of exoCDN2 with increasing concentrations of unloaded EVs (n = 2 biological replicates per donor). **g, h** Dendritic cell and monocyte activation in human PBMC were measured after treating with free CDN1 and exoCDN1 (n = 12 healthy donors). Representative dose-response curves of CD86 MFI and C_{max} value of CD86 MFI on cDC (**g**) and monocytes (**h**) (n = 2 biological replicates per donor). **, P < 0.01; ****, P < 0.0001 by unpaired t-test. Data are presented as means ± s.d. from replicate samples as indicated.

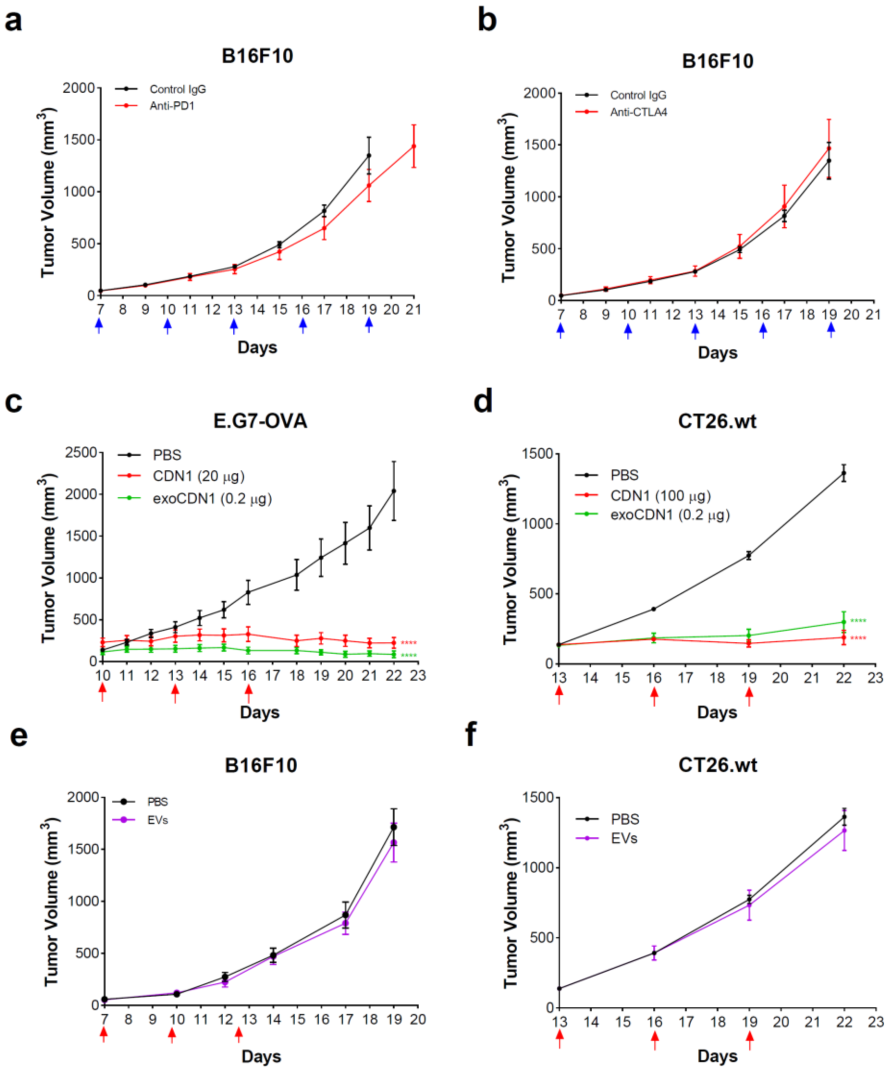

Supplementary Fig. 4: *In vivo* tumor growth measurement. a, b B16F10 tumor growth were measured over time (n = 5 animals per group) after injection of IgG (10 mg/kg), anti-PD1 (10 mg/kg), or anti-CTLA4 (5 mg/kg) intraperitoneally 3 times with 3 days interval. **c, d** EG7-OVA tumor (n = 5 animals per group) (**c**) and CT26.wt tumor (n = 7 animals per group) (**d**) growth was measured over time after 3 IT dosing of PBS, CDN1, or exoCDN1. ****, $P < 0.05$ by two-way ANOVA with Tukey's multiple comparison test. **e, f** B16F10 tumor (n = 5 animals per group) (**e**) and CT26.wt tumor (n = 7 animals per group) (**f**) growth was measured over time after 3 IT dosing of PBS or unloaded EVs. Red and blue arrows in the graph indicate IT injection days and intraperitoneal injection days, respectively. Data are presented as means ± s.e.m from replicate samples as indicated.

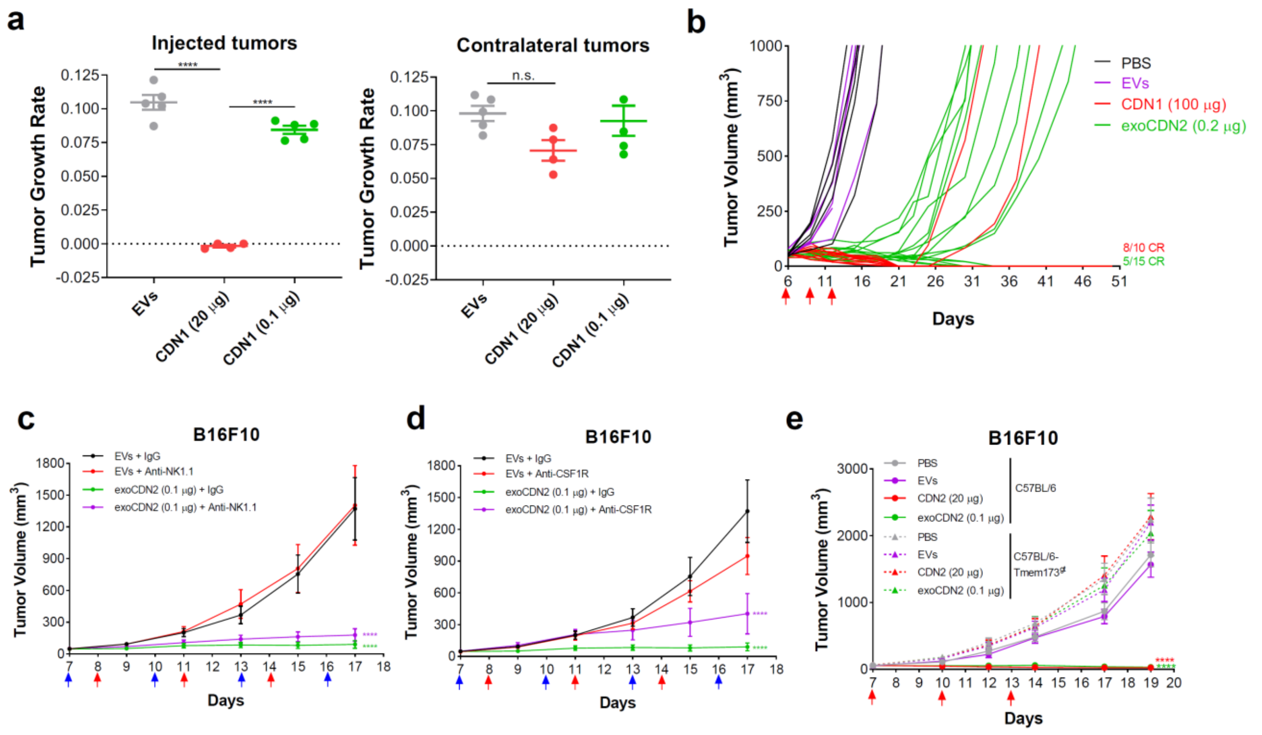

Supplementary Fig. 5: Systemic anti-tumor activity by exoSTING via T-cell and STING. a Growth rates of B16F10 tumor cells implanted subcutaneously in right (1×10^6 cells) and left (5×10^5 cells) flanks of mice (*n* = 5 animals for PBS and CDN1 (0.1 µg); *n* = 4 animals for CDN1 (20 µg)). Unloaded EVs, 0.1 or 20 µg of CDN1 were injected intratumorally into right flank tumors. Both injected and non-injected contralateral tumor growth was measured, and tumor growth rate was calculated. ****, $P < 0.05$ by one-way ANOVA with Tukey's multiple comparison test. **b** Individual plots of subcutaneous B16F10 tumors treated with PBS (*n* = 5 animals), unloaded EVs (*n* = 5 animals), 100 µg of CDN1 (*n* = 10 animals), and exoCDN2 (0.2 µg) (*n* = 15 animals) intratumorally 3 times with 3 days interval. **c, d** Unloaded EVs or exoCDN2 were injected into subcutaneous B16F10 tumors intratumorally 3 times with 3 days interval. Red arrows in the graph indicate IT injection days. B16F10 tumor bearing mice received the IgG (10 mg/kg), anti-NK1.1 antibody (10 mg/kg) (**c**), or anti-CSF1R antibody (10 mg/kg) (**d**) intraperitoneally, one day before IT injection (*n* = 5 animals per group). **e** Tumor growth inhibition in C57BL/6-Tmem173^{gt} (STING knockout mice) (*n* = 5 animals per group). Both C57BL/6 and C57BL/6-Tmem173^{gt} mice were implanted subcutaneously with 1×10^6 B16F10 cell on right flank of mice. PBS, unloaded EVs, CDN2 (20 µg), exoCDN2 (0.1 µg) were injected intratumorally. Red and blue arrows in the graph indicate IT injection days and intraperitoneal injection days, respectively. ****, $P < 0.05$ by two-way ANOVA with Tukey's multiple comparison test. Data are presented as means ± s.e.m from replicate samples as indicated.

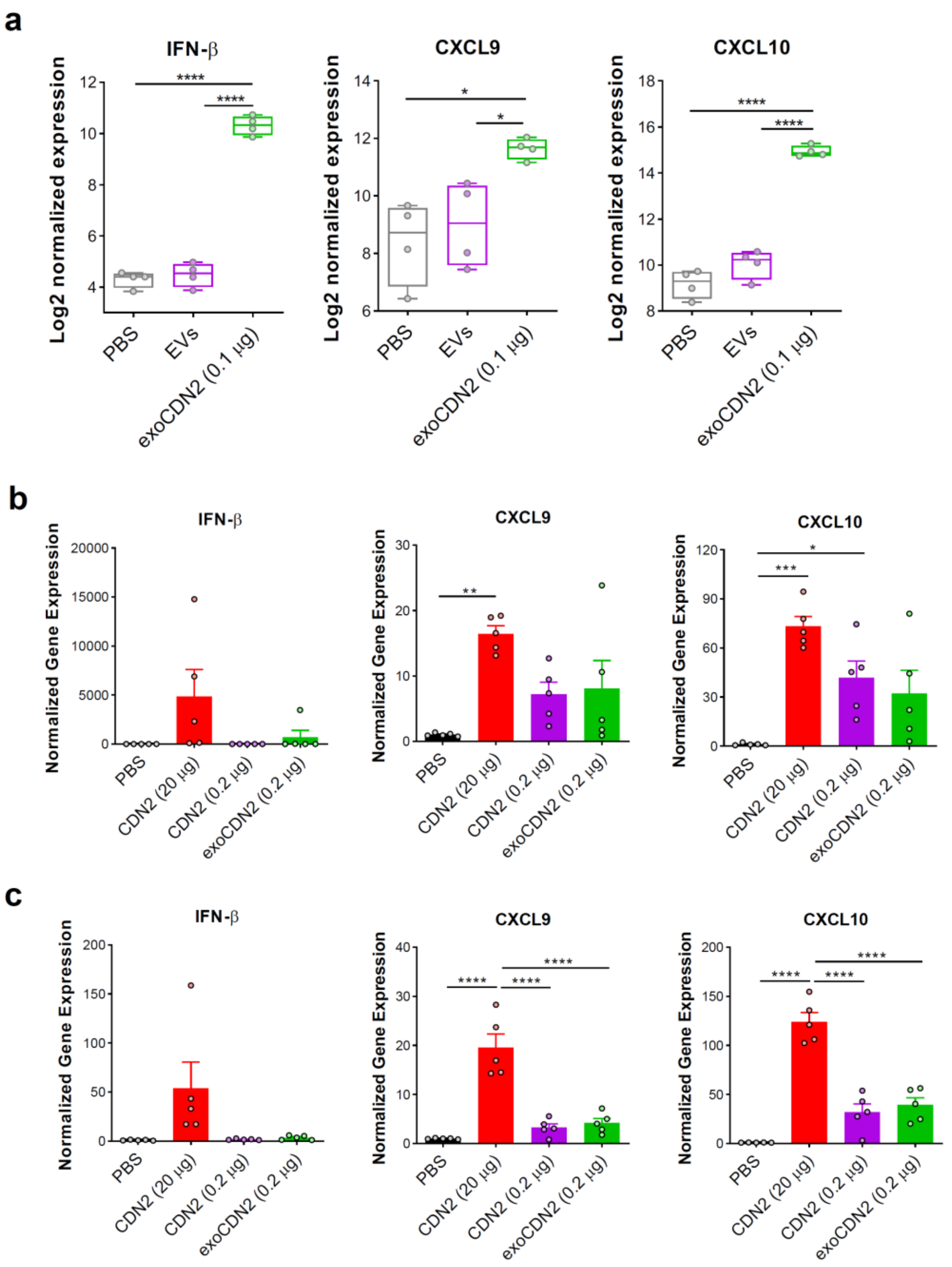

Supplementary Fig. 6: exoSTING did not activate STING pathway in distal organs. a Normalized expression level of IFN-β, CXCL9, and CXCL10 4 h after 1 IT injection of PBS, unloaded EVs, and exoCDN2 (0.1 µg) into B16F10 tumors, analyzed by Nanostring. **b, c** Tumor draining lymph nodes and spleens were collected after IT dosing as a part of study described in Fig. 3 c-e. Briefly, four h after IT injection to B16F10 tumor, RNAs were purified from tumor draining lymph nodes and spleens (n = 5 animals per group). Relative expression of IFN-β, CXCL9, and CXCL10 genes in tumor draining lymph nodes (**b**) and spleens (**c**) was measured by RT-qPCR, normalized against housekeeping gene *RPS13*. Data are presented as means ± s.e.m from individual biological replicate samples. *, $P < 0.05$; **, $P < 0.01$; ***, $P < 0.001$; ****, $P < 0.0001$ by one-way ANOVA with Tukey's multiple comparison test.

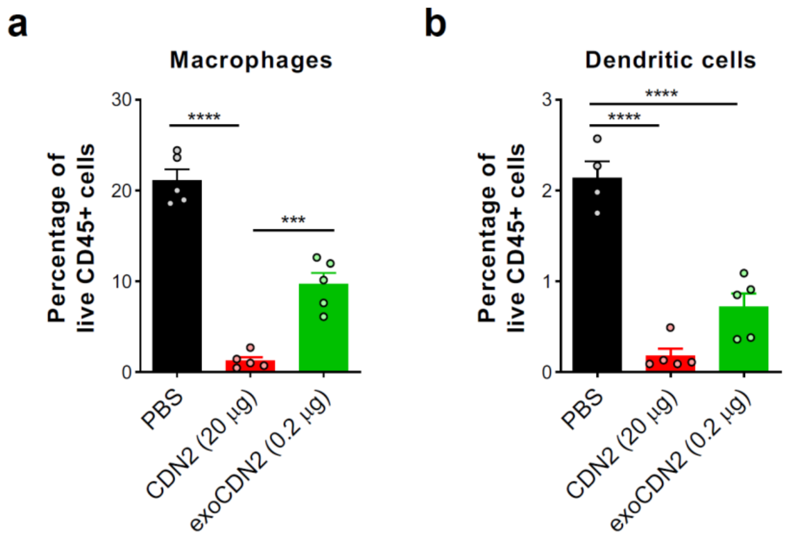

Supplementary Fig. 7: Free CDN induced immune cell ablation in tumor TME. Percentage of macrophages (**a**) and dendritic cells (**b**) in live $CD45^+$ cells in tumors were measured by flow cytometry after 2 doses of PBS, CDN2 (20 µg), and exoCDN2 (0.2 µg) into B16F10 tumors (n = 5 animals per group). Data are presented as means ± s.e.m from individual biological replicate samples. ***, $P < 0.001$; ****, $P < 0.0001$ by one-way ANOVA with Tukey's multiple comparison test.

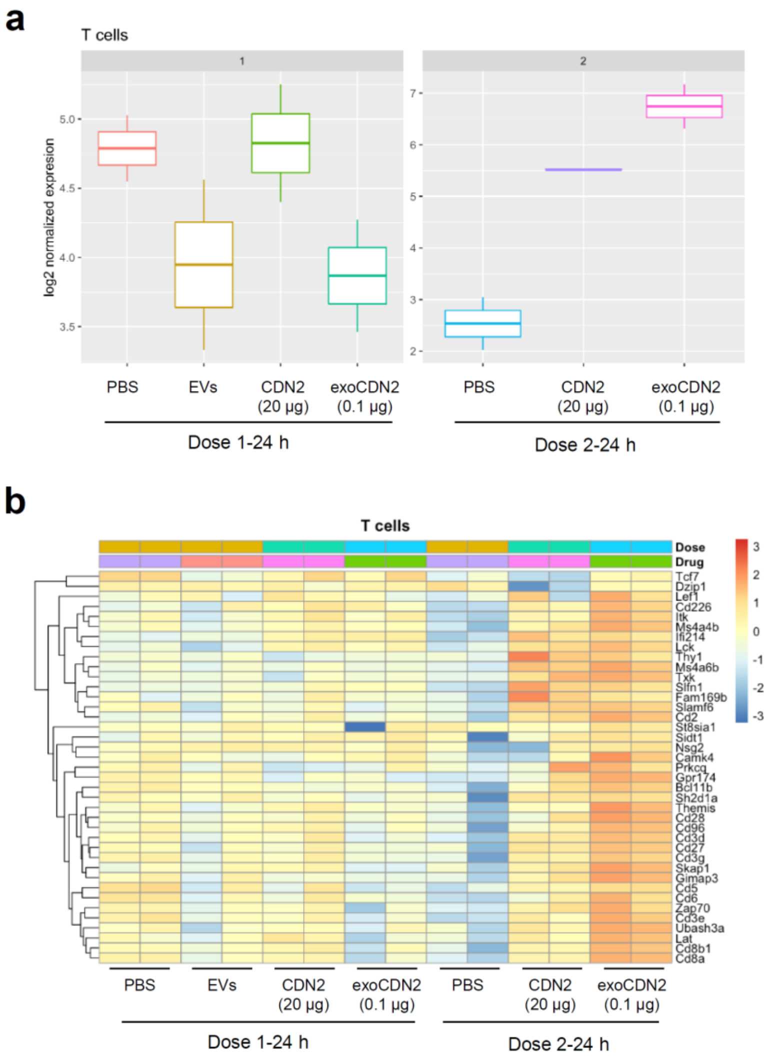

Supplementary Fig. 8: exoSTING induced interferon stimulated gene signatures. Normalized expression level (**a**) and heatmap (**b**) of T cell related genes 24 h after 1 or 2 IT injections of PBS, unloaded EVs, CDN2 (20 µg) or exoCDN2 (0.1 µg) into B16F10 tumors (*n* = 2 biological replicates per group), analyzed by RNA sequencing. The data is representative of 5 of biological replicate samples in each treatment group.

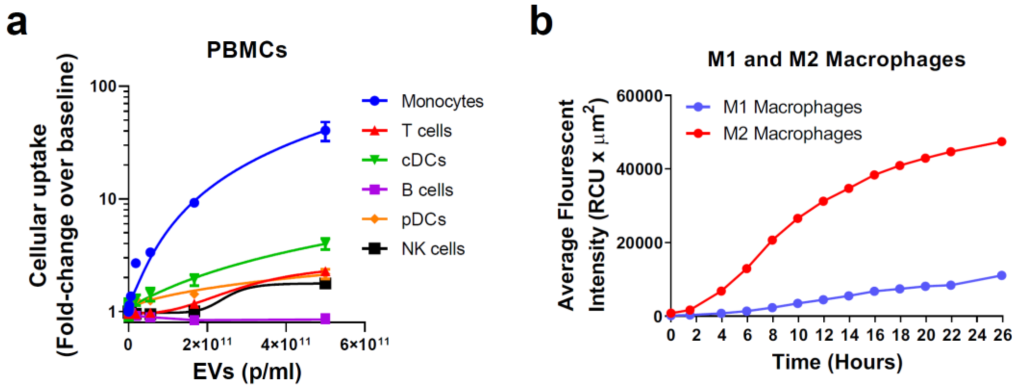

Supplementary Fig. 9: Uptake of EVs in different immune cells. a Uptake of PTGFRN$^{+/+}$ EVs that tagged with GFP in different subsets of human PBMC after overnight incubation (*n* = 4 healthy donors). **b** Uptake of PTGFRN$^{+/+}$ EVs that are labeled with pHrodoRed dye in human M1 or M2 macrophages over time (*n* = 3 healthy donors). Data are presented as means ± s.e.m from individual biological replicate samples.

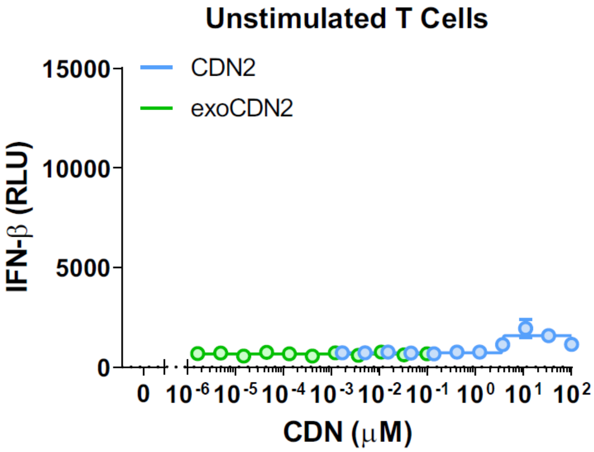

Supplementary Fig. 10: exoSTING has no effect on T cells. Representative dose-response curves (n = 3 healthy donors) of IFN-β production in unstimulated naïve T cells after treating with free CDN2 and exoCDN2 (n = 2 biological replicates per donor). Data are presented as means ± s.d. from individual biological replicate samples.

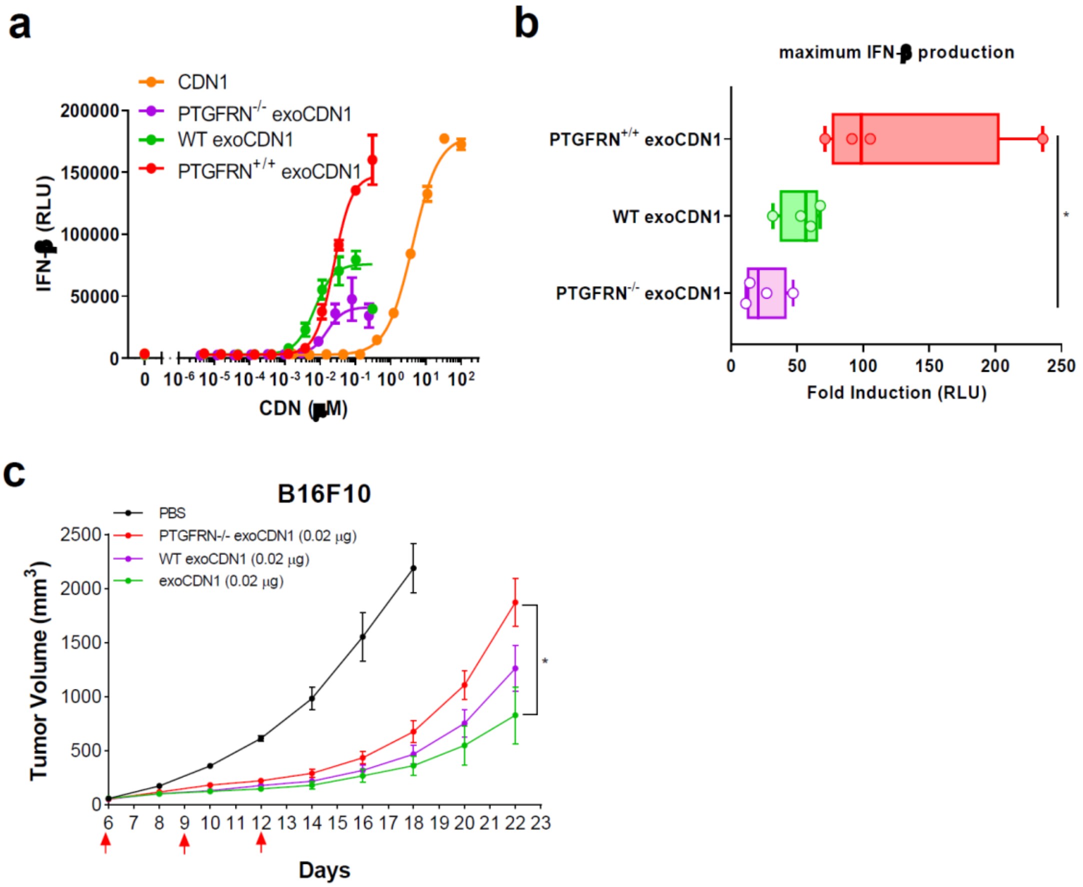

Supplementary Fig. 11: PTGFRN on EVs enhances activity of exoSTING. a Representative dose-response curves (*n* = 4 healthy donors) of IFN-β production in human PBMC supernatant after incubating with PTGFRN$^{-/-}$ exoCDN1, WT exoCDN1, and PTGFRN$^{+/+}$ exoCDN1 (*n* = 2 biological replicates per donor). **b** Fold induction of maximum IFN-β production over background were calculated after incubating with PTGFRN$^{-/-}$ exoCDN1, WT exoCDN1, and PTGFRN$^{+/+}$ exoCDN1 (*n* = 4 healthy donors). **c** B16F10 tumor growth was measured after intratumor injection of PTGFRN$^{-/-}$ exoCDN1, WT exoCDN1, and PTGFRN$^{+/+}$ exoCDN1 (*n* = 5 animals per group). *, $P < 0.05$ by two-way ANOVA with Tukey's multiple comparison test. Data are presented as means ± s.e.m from individual biological replicate samples.

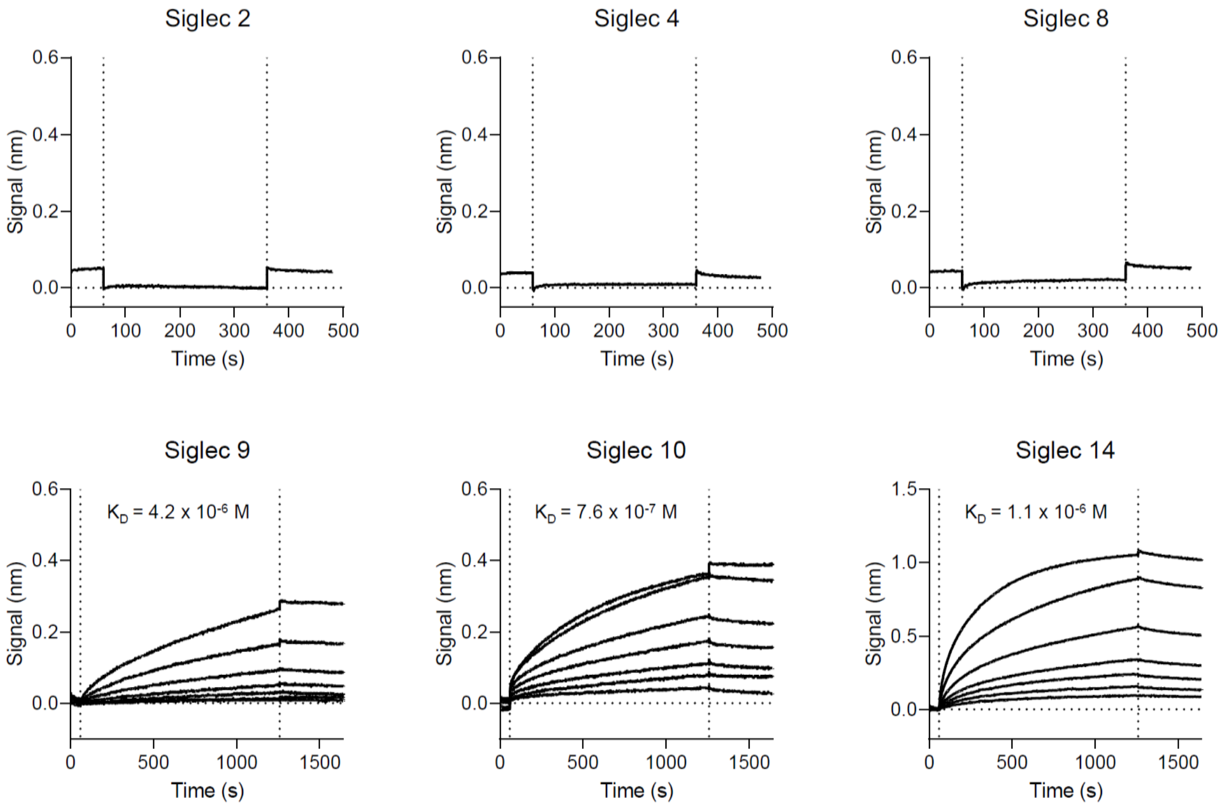

Supplementary Fig. 12: PTGFRN binds to select Siglec proteins. Biolayer interferometry analysis of binding interactions between the extracellular domain of PTGFRN and Siglec 2, 4, 8, 9, 10, and 14. Fc-fused Siglec proteins (5 µg/mL) were immobilized on Protein A biosensors and incubated with 2-fold serial dilutions of PTGFRN starting at 100 µg/mL. Equilibrium dissociation constants were calculated for Siglec 9, 10, and 14 using ForteBio Data Analysis software v. 10.0. Baseline, association, and dissociation phases are separated by the vertical dotted lines.

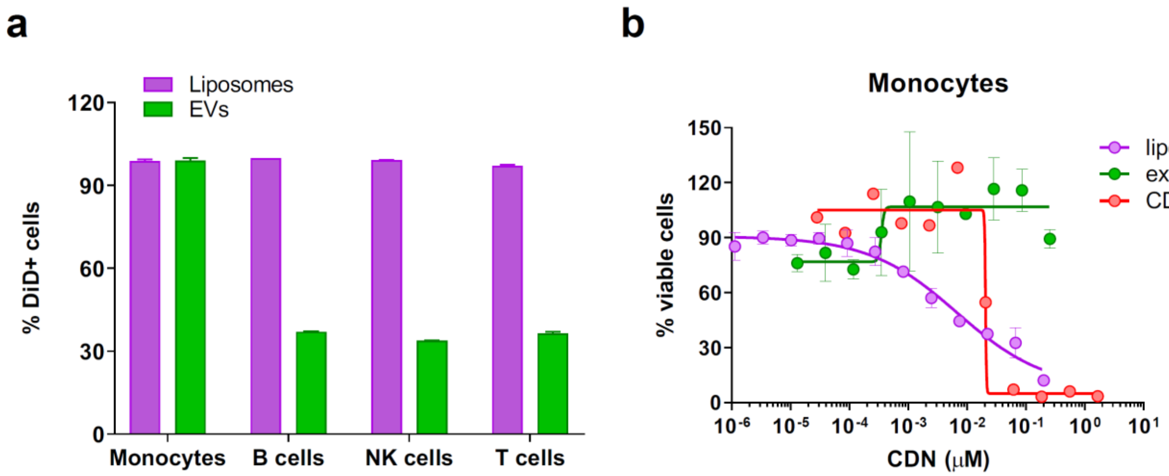

Supplementary Fig. 13: exoSTING preserved viability of APC. a Uptake of DiD labeled liposomes or EVs in different immune cells in PBMCs (n = 2 biological replicates). **b** Representative dose-response curves (n = 2 healthy donors) of monocyte viability *in vitro* after incubating with exoCDN1, lipoCDN1, and free CDN1 (n = 2 biological replicates per donor). Data are presented as means ± s.d from individual biological replicate samples.

a

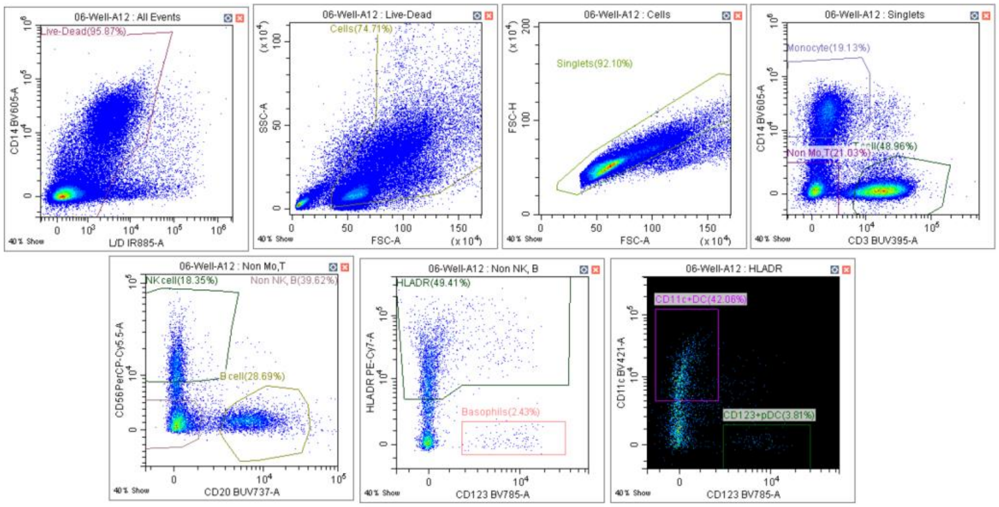

b

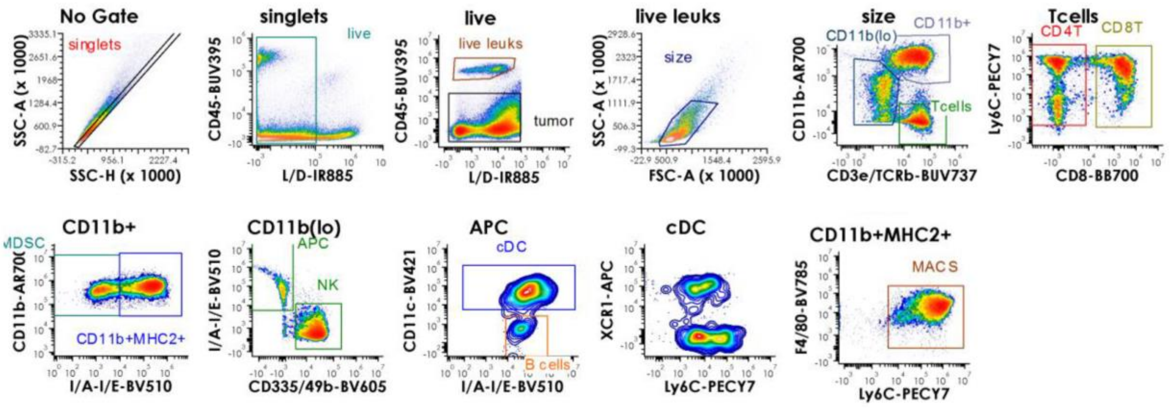

Supplementary Fig. 14: Gating strategies for flow cytometry. a Gating strategy for human PBMC assays for Supplementary Fig. 3; **b** Gating strategy for mouse tumors for Fig. 4d, f and Supplementary Fig. 7.

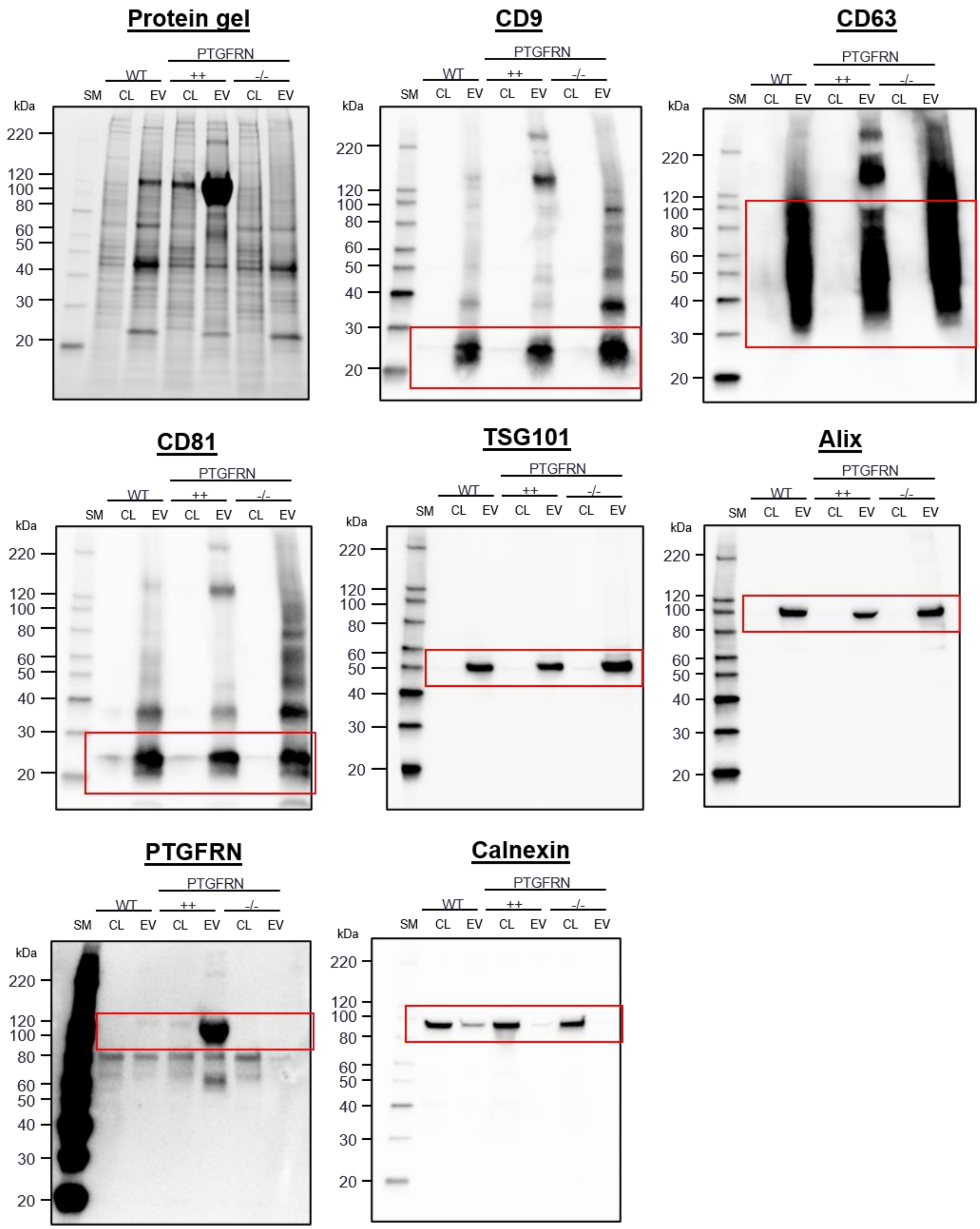

Supplementary Fig. 15: Full Western Blot images. Whole gel images that are used in Supplementary Figure 1d. SM; size marker, CL: cell lysate, EV; extracellular vesicles. Red squares represent cropping areas.
