# Supplementary material for: ExoSTING, an extracellular vesicle loaded with STING agonists, promotes tumor immune surveillance
Source: Commun Biol. 2021 Apr 22;4:497. doi: 10.1038/s42003-021-02004-5 (PMC8062530; doi:10.1038/s42003-021-02004-5)
Supplement: Supplementary file 5 — Reporting Summary [file 42003_2021_2004_MOESM5_ESM.pdf]

nature research

Corresponding author(s): Sriram Sathyanarayanan
Last updated by author(s): Mar 12, 2021

Reporting Summary

Nature Research wishes to improve the reproducibility of the work that we publish. This form provides structure for consistency and transparency in reporting. For further information on Nature Research policies, see our Editorial Policies and the Editorial Policy Checklist.

Statistics

For all statistical analyses, confirm that the following items are present in the figure legend, table legend, main text, or Methods section.

n/a	Confirmed	
☐	☒	The exact sample size (*n*) for each experimental group/condition, given as a discrete number and unit of measurement
☐	☒	A statement on whether measurements were taken from distinct samples or whether the same sample was measured repeatedly
☐	☒	The statistical test(s) used AND whether they are one- or two-sided<br>*Only common tests should be described solely by name; describe more complex techniques in the Methods section.*
☐	☒	A description of all covariates tested
☒	☐	A description of any assumptions or corrections, such as tests of normality and adjustment for multiple comparisons
☐	☒	A full description of the statistical parameters including central tendency (e.g. means) or other basic estimates (e.g. regression coefficient) AND variation (e.g. standard deviation) or associated estimates of uncertainty (e.g. confidence intervals)
☐	☒	For null hypothesis testing, the test statistic (e.g. *F*, *t*, *r*) with confidence intervals, effect sizes, degrees of freedom and *P* value noted<br>*Give P values as exact values whenever suitable.*
☒	☐	For Bayesian analysis, information on the choice of priors and Markov chain Monte Carlo settings
☒	☐	For hierarchical and complex designs, identification of the appropriate level for tests and full reporting of outcomes
☒	☐	Estimates of effect sizes (e.g. Cohen's *d*, Pearson's *r*), indicating how they were calculated

Our web collection on statistics for biologists contains articles on many of the points above.

Software and code

Policy information about availability of computer code

Data collection	Flow cytometry data was collected using Cyto Flex LX (Beckman Coulter) or SA3800 (Sony). SDS-PAGE and Western blot data were collected using Compass Software (v. 3.1.8, Protein Simple) or Image Lab (v. 6.0.1, BioRad). Particle counting was performed using Nanosight NTA software (v. 3.4, Malvern Panalytical). Elispot data was acquired using Elispot Reader software (v. 7.0, Autoimmun Diagnostika GMBH). Nanostring data was acquired using nCounter Sprint Profiler (Nanostring). qPCR data was acquired using QuantStudio™ 3 & 5 Real-Time PCR System (Thermo Fisher Scientific). Binding of PTGFRN and Siglec proteins were measusred by using Protein A biosensors (ForteBio).
Data analysis	Statistical analyses and graph generation were performed using Prism (v. 8.1, Graphpad). Flow cytometry data was processed using FlowJo (v. v10.6.1; Becton, Dickinson & Company) or FCS Express (v. 7, De Novo Software). Nanostring data was analyzed using nSolver Analysis Software (v 4.0, Nanostring). qPCR data was analyzed using QuantStudio™ Design & Analysis Software (v. 1.5.1 Thermo Fisher Scientific). Equilibrium dissociation constants were calculated for Siglec 9, 10, and 14 using ForteBio Data Analysis software v. 10.0.

For manuscripts utilizing custom algorithms or software that are central to the research but not yet described in published literature, software must be made available to editors and reviewers. We strongly encourage code deposition in a community repository (e.g. GitHub). See the Nature Research guidelines for submitting code & software for further information.

Data

Policy information about availability of data

All manuscripts must include a data availability statement. This statement should provide the following information, where applicable:

- Accession codes, unique identifiers, or web links for publicly available datasets
- A list of figures that have associated raw data
- A description of any restrictions on data availability

Data supporting the findings of this work are available within the paper and its Supplementary files. RNA sequencing data have been deposited to GEO with

accession number GSE168784. Source data is provided in Supplementary Data 1. All data are available upon reasonable request.

Field-specific reporting

Please select the one below that is the best fit for your research. If you are not sure, read the appropriate sections before making your selection.

- [x] Life sciences
- [ ] Behavioural & social sciences
- [ ] Ecological, evolutionary & environmental sciences

For a reference copy of the document with all sections, see nature.com/documents/nr-reporting-summary-flat.pdf

Life sciences study design

All studies must disclose on these points even when the disclosure is negative.

Sample size	All experimental measurements were performed at least in duplicate, and where feasible, sample sizes are listed in the manuscript. All immune cells assays relied on multiple donors, and all animal studies used at least five animals per group.
Data exclusions	No data were excluded.
Replication	All attempts at replication were successful.
Randomization	Mice were implanted with tumor cells until the tumors reached an average volume of 50-100mm^3. Mice were randomized into groups such that the average tumor volume and variance of tumor volume between individual animals was as consistent as possible. Groups were treated as described in the materials and methods.
Blinding	No experiments were blinded, since all animal study read outs were objective.

Reporting for specific materials, systems and methods

We require information from authors about some types of materials, experimental systems and methods used in many studies. Here, indicate whether each material, system or method listed is relevant to your study. If you are not sure if a list item applies to your research, read the appropriate section before selecting a response.

Materials & experimental systems

n/a	Involved in the study	
	x	Antibodies
	x	Eukaryotic cell lines
x		Palaeontology and archaeology
	x	Animals and other organisms
x		Human research participants
x		Clinical data
x		Dual use research of concern

Methods

n/a	Involved in the study	
x		ChIP-seq
	x	Flow cytometry
x		MRI-based neuroimaging

Antibodies

Antibodies used

Human anti-CD9 BioLegend 312102
Human anti-CD63 ThermoFisher 10628D
Human anti-CD81 ThermoFisher 10630D
Human anti-TSG101 BD Biosciences 612696
Human anti-Alix Abcam ab117600
Human anti-Calnexin Abacam ab22595
Human anti-PTGFRN LS-Bio LS-C158765
Anti-Adenovirus Type 5 E1A antibody Abcam ab204123
Rabbit anti-Mouse IgG HRP Abcam ab97046
Goat anti-Rabbit IgG HRP Abcam ab6721

Anti-mouse CD45 BUV395 BD Biosciences 564279
Anti-mouse CD3e BUV737 BD Biosciences 564618
Anti-mouse TCRb BUV737 BD Biosciences 564799
Anti-mouse CD11b APC BioLegend 101212
Anti-mouse CD8 BB700 BD Biosciences 566409
Anti-mouse Ly-6C PE-Cy7 BioLegend 128018
Anti-mouse I-A/I-E BV510 BioLegend 107635
Anti-mouse CD335 BV605 BioLegend 137619

Anti-mouse CD49b BV605 BD Biosciences 740363
Anti-mouse CD11c BV421 BioLegend 117330
Anti-mouse XCR1 APC BioLegend 148206
Anti-mouse F4/80 BV785 BioLegend 123141

Anti-mouse CD8 Abcam ab230156
Anti-mouse F4/80 Cell Signaling 70076s
Anti-mouse phospho TBK1 Cell Signaling 5483
Anti-mouse Cleaved caspase 3 Cell Signaling 9661

Anti-human CD69 PE-Cy7 BioLegend 310912
Anti-human CD86 APC BioLegend 305412
Anti-human CD11c BV421 BioLegend 301628
Anti-human CD1C BV711 BioLegend 331536
Anti-human CD14 BV605 BioLegend 367126
Anti-human CD123 BV785 BioLegend 306032
Anti-human CD141 PE-Cy7 BioLegend 344110
Anti-human CD3 BUV395 BD Biosciences 564117
Anti-human CD19 APC BioLegend 302212
Anti-human CD20 BUV737 BD Biosciences 564432
Anti-human CD16 PE-Cy7 BioLegend 302016
Anti-human CD56 PerCP-Cy5.5 BioLegend 318326
Anti-human HLADR PE BioLegend 307606

Validation

All antibodies were validated by the manufacturer, when applicable. PTGFRN antibodies were validated against recombinant PTGFRN by biolayer interferometry and by testing against PTGFRN knock out samples.

Eukaryotic cell lines

Policy information about cell lines

Cell line source(s)

HEK293SF cells were obtained under license from NRCC-CNRC. All other cell lines were obtained from ATCC.

Authentication

All cell lines were authenticated by the vendor including tests for identity, species, and presence of pathogenic viruses. No additional authentication was carried out.

Mycoplasma contamination

Cells were tested for Mycoplasma and were negative.

Commonly misidentified lines
(See ICLAC register)

No commonly misidentified cell lines were used in this study.

Animals and other organisms

Policy information about studies involving animals; ARRIVE guidelines recommended for reporting animal research

Laboratory animals

All mice used in the study were female C57BL/6 mice , female BALB/c mice, or female female BALB/cAnNHsd mice, aged five to ten weeks, that were obtained from Taconic, The Jackson Laboratory, or Envigo, respectively.

Wild animals

No wild animals were used

Field-collected samples

No field-collected samples were used

Ethics oversight

Codiak’s internal IACUC and the Cambridge Animal Commissioner approved study protocols and provided guidance on the care and ethical treatment of all study animals.

Note that full information on the approval of the study protocol must also be provided in the manuscript.

Flow Cytometry

Plots

Confirm that:

- [x] The axis labels state the marker and fluorochrome used (e.g. CD4-FITC).
- [x] The axis scales are clearly visible. Include numbers along axes only for bottom left plot of group (a 'group' is an analysis of identical markers).
- [x] All plots are contour plots with outliers or pseudocolor plots.
- [x] A numerical value for number of cells or percentage (with statistics) is provided.

Methodology

Sample preparation	Preparation of samples is detailed in the materials and methods.
Instrument	Beckman CytoFLEX and Sony SA3800
Software	Flow cytometry data was collected using CytExpert (v. 2.3.0.84, Beckman Coulter) or SA3800 (v. 2.0.4.13263, Sony)
Cell population abundance	All measured cell populations were pure and abundant (>1% of analyzed population) as determined by the flow cytometry plots
Gating strategy	All flow cytometry plots were gated on live single cells and all gating boundaries were based on unstained controls, isotype controls, and FMOs. See Supplemental Figure 17 for exemplary gating strategies.

☒ Tick this box to confirm that a figure exemplifying the gating strategy is provided in the Supplementary Information.
